# Outcomes from the ASPEN intervention program: a randomized clinical trial of a culturally adapted parent-mediated intervention program in low-resource settings

**DOI:** 10.3389/fpsyt.2026.1795918

**Published:** 2026-06-19

**Authors:** Sandra B. Vanegas, Sandy Magaña

**Affiliations:** School of Social Work, University of Texas at Austin, Austin, TX, United States

**Keywords:** autism, children, families, low-resource, parent-mediated intervention

## Abstract

This randomized controlled study examined the efficacy of a culturally adapted parent-mediated intervention for parents and young children with autism (*n* = 45) from low-resource households (ClinicalTrials.gov Identifier: NCT04505488). Families from these settings are often left out of autism intervention studies and arguably have unique needs not considered in autism intervention work. Parent/child dyads were randomized to the intervention group, which received 12 weekly sessions that included psychoeducation and coaching of evidence-based (EB) strategies with their children, or the comparison group who received all materials and 4 triweekly check-in sessions. We found that parents in the intervention group showed improvement in their self-efficacy (confidence) in using EB strategies, compared to the comparison group, and both groups improved in the use of EB strategies. Parents in the comparison group also improved in feeling more empowered and their children improved in social communication skills. These findings suggest that less intense parent interventions may be just as efficacious as those that require more regular participation for families from low-resource households. More research is needed to find the right balance and active ingredients to benefit these families.

**Clinical Trial Registration:**

https://clinicaltrials.gov/study/NCT04505488, identifier NCT04505488.

## 1 Introduction

Numerous national and public health data find that children from low-resource households and communities are less likely to be identified as having autism, are less likely to be identified in early childhood (< 5 years of age), and are less likely to receive evidence-based treatments (1–5). Several factors have been identified as contributing to these disparities, including provider biases, access to healthcare, insurance, availability of providers, availability of interpreters, and transportation (6, 7). Low socioeconomic resources may further exacerbate these challenges for families of children with autism. For example, families with limited financial resources may have unstable housing, inadequate insurance coverage, food insecurity, and reside in neighborhoods with high levels of risk factors and low levels of protective factors (8). Families of children with autism with limited financial resources may prioritize safety and basic needs above other demands, including devoting significant time and effort into intensive therapies for their child with autism (9). For this paper, we use the person-first language as that was the preferred term used by caregivers in this study. For families with low resources, the treatment recommendations and costs may not be feasible, practical or sustainable given the required investment of time, money, and effort. Parent-mediated programs (PMI) where parents are provided education, coaching, and support can be a viable mechanism to address the need for more family-centered evidence-based approaches to caring for children with autism in low-resource communities.

### 1.1 Parent mediated intervention programs

PMI programs empower and train parents to be the agents of change by teaching them specific strategies and techniques to support and improve their child’s developmental outcomes (10). PMI programs can teach parents on a range of topics, from communication to challenging behavior, through didactics, coaching, and practice with supervision (11). The rationale for PMI programs draws upon the important role that parents have on the developmental trajectories of their children (12). For example, parents have invaluable knowledge about their child’s behavior and challenges, have consistent access to their child, and can implement parenting practices across contexts and settings for greater generalization of skill development. These factors have been identified as key in the success of early intervention programs for children with autism (13). A subset of PMI programs are based on naturalistic, developmental behavioral intervention (NDBI) principles that recognize the value of providing social models within the child’s proximal range of development in natural settings and contexts (14, 15). NDBI programs combine both behavioral and developmental strategies through specific instructional approaches (e.g., environmental arrangement, balanced turns, modeling, child-led activities, natural reinforcement) to support gains in development (16). Incorporating psychoeducation within NDBI-based PMI programs provides further support for the learning and implementation of evidence-based strategies to support children’s development. Psychoeducation is particularly critical for programs that want to reach and engage parents and caregivers with high levels of anxiety, depression, and stress (17). The use of PMI programs that empower parents with evidence-based strategies can also address the limited availability of health care service providers with adequate training, knowledge, and skills to care for children with autism (18). For example, many families with limited financial resources or who live in rural communities, often wait months or years to gain access to services and therapies (19–21). Families who receive PMI training of evidence-based strategies can therefore fill their child’s service needs while on waitlists for services or therapists.

Raising a child with autism or a developmental disability requires parents and caregivers to become strong advocates for their children across many areas, including education, healthcare, and community living (22). Families from low-resource households may experience an even greater need to advocate for their children, as evidenced by the vast literature on disparities in access to services (23, 24). Thus, PMIs can fill in many gaps that families in low-resource households face with access to evidence-based supports. For example, recent systematic reviews have found significant positive effects of receiving PMIs on parent-reported child adaptive behavior, child social communication skills, parent-child relationships (25–30). Despite a significant body of research findings suggesting the positive impact on child and parent outcomes (31), few research studies have focused on families in low-resource households (32–34). To fill these significant needs, our team set out to develop a parent-mediated intervention designed to be responsive to the needs of low-resource communities.

### 1.2 Development of the ASPEN intervention program

Based on the growing evidence of PMIs in supporting children with autism and their families, and the limited research focusing on low-resource communities, we set out to test the efficacy of a culturally adapted PMI, the ASD Screening and Parent ENgagement (ASPEN) intervention program (35, 36). The ASPEN intervention program was based on two evidence-based intervention programs, *Parents Taking Action* (37) and *Project ImPACT* (38, 39) that had demonstrated positive outcomes for parents of children with autism (35, 37, 40–43). The initial development of the ASPEN intervention program involved a comparison of the content and format of the Parents Taking Action and the Project ImPACT manuals. Similar sections were combined and updated, and new content was also added (e.g., developmental milestone resources, learning two or more languages). Subsequent adaptation followed an iterative approach with a development phase where the intervention strategies (e.g., content, examples, visuals) were delivered and implemented with two families of young children with autism. Parents reviewed materials (i.e., session summaries) and provided feedback on the feasibility, acceptability, and usability of the intervention strategies, delivery formats, and materials. Two pilot trials followed, one in English and one in Spanish, which tested the feasibility of group and individual session implementation. Feedback from these pilot trials informed the final development of the curriculum (e.g., reorganization of sessions to support adult learning) and design of the intervention (e.g., individual sessions to allow for more flexibility). For more details, readers are referred to prior work outlining the development and adaptation of the ASPEN Intervention Program (35, 36).

The current study set out to examine the efficacy of the ASPEN Intervention Program in supporting families in low-resource households. We compared two delivery models of the culturally adapted PMI, 1) a 12-session model delivered by a clinician and peer leader versus 2) a 4-session model delivered by a clinician, and their effects on child (adaptive behavior, challenging behavior, social communication) and parent/family outcomes (parenting stress, family empowerment, family functioning, intervention strategy efficacy and use).

## 2 Methods

### 2.1 Study design

We used a stratified block randomization approach to ensure balance between group assignments across the participants’ primary language. We used a simple randomization approach within each language (English, Spanish). The randomization sequences were generated using an Excel random number generator, with group assignments within each stratum determined upon enrollment and initiation of the baseline assessment. The allocation sequence was uploaded into the randomization module of REDCap, which would populate the group assignment on screen to the research assistant upon completion of the baseline assessment. Research assistants who conducted the baseline and post-intervention assessments were blind to the participants’ group assignment during the administration of the assessments.

### 2.2 Participants

The study population included young children between 18 months and 6 years of age and their primary caregiver who lived in Texas. To be eligible, the child had to have a prior diagnosis or educational classification of autism, or if no diagnosis or educational classification was available, then the child must have a high likelihood of autism based on autism screening tools (listed below). Additionally, the caregiver and child must reside in a low-resource household defined as 1) having a primary caregiver with a high school or lower education, 2) have Medicaid as a primary health insurance, or 3) have a household income meeting or below the 200% Federal Poverty Level criteria based on household income and the number of persons living in the household (33). Participant recruitment began in February 2020 and continued through April 2025.

### 2.3 Variables and instruments

#### 2.3.1 Autism screening tools

##### 2.3.1.1 Modified checklist for autism in toddlers-revised with follow-up interview

This measure contains 20 questions describing specific developmental characteristics (44). It is a widely used screening instrument to assess likelihood for autism among young children. If the child fails 3 or more items, they are considered to have a high likelihood of autism. Recent data on the M-CHAT-R/F finds adequate internal consistency (α = 0.79), high sensitivity (0.91), and high specificity (0.95) (44). The M-CHAT-R/F was also available in Spanish. We administered the M-CHAT-R to children aged between 18 to 47 months who did not have a prior diagnosis or educational classification of autism.

##### 2.3.1.2 Social communication questionnaire: lifetime version

This measure has been used extensively to screen children for autism who are aged 4 years and older (45). Questions capture a range of behaviors, including reciprocal social interaction skills, communication, and restricted, repetitive, and stereotyped patterns of behavior. For eligibility purposes, we used a cutoff score of 12 if there were other indications of developmental delays or developmental concerns. The SCQ has strong sensitivity (0.90) and specificity (0.72) in accurately identifying children with autism (46). The SCQ has also been translated in Spanish (47). This measure was only administered to children aged between 30 to 83 months who did not have a prior autism diagnosis or educational classification.

For children ages 30 months through 47 months, we administered both the M-CHAT-R and the SCQ-Lifetime. To confirm eligibility, the child had to meet the respective measure’s cutoff for at least one screening to qualify. Given that our focus was not to confirm an autism diagnosis, but rather, confirm whether the child had a high likelihood of autism, the administration of the M-CHAT-R/F and the SCQ-Lifetime would be sufficient. Other studies have also confirmed the use of these screening tools to confirm autism diagnoses as pathways for enrollment in intervention studies and autism registries (48–50).

#### 2.3.2 Demographic information

A project-specific demographic questionnaire was developed to collect information regarding the child’s diagnoses, language exposure, and access to community-based services (i.e., receipt of service, if yes, number of minutes received per week). Additionally, the primary caregiver was asked about their nativity, level of education, language proficiencies, employment status, and household income.

#### 2.3.3 Child profiles

##### 2.3.3.1 Vineland adaptive behavior scales – 3rd edition (VABS-3)

This parent/caregiver interview/rating form measure has been validated for diverse groups of children and will provide a parent/caregiver perspective on the child’s adaptive behavior in daily settings (51). Three domains were assessed: Communication, daily living skills, and socialization. An overall adaptive behavior composite score was also obtained. The VABS-3 interview has good internal consistency (α = .90 -.98) and good test-retest reliability (*r* = .79 -.89). The VABS-3 has also been validated for use in Spanish with the Caregiver Rating Form. The interview form was used for assessments completed in English, whereas the caregiver rating form was used for assessments completed in Spanish. The VABS-3 Adaptive Behavior Composite standard score served as the primary child outcome, with higher scores indicating greater adaptive behavior skills. For VABS-3 subscales, growth scale values were used in analyses as past studies have shown that these scores are more sensitive to change (52, 53).

##### 2.3.3.2 Scales of independent behavior-revised problem behavior subscale

The SIB-R measured children’s challenging behaviors. Parents/caregivers were asked to rate the frequency and severity of behavior problems during the past 6 months (54). The SIB-R has adequate test-retest reliability (*r* = .83 -.97). The Problem Behavior subscale has been translated in Spanish previously and has been used with Latino, Spanish-speaking families of children with disabilities (37, 55). The sum of problem behaviors endorsed by the caregiver was used as a secondary child outcome (37).

##### 2.3.3.3 Social communication questionnaire: current version

The SCQ Current version (45), similar to the Lifetime version, is designed to capture social communication skills among individuals. Although the Lifetime version is designed to screen for autism, the Current version is adequate for measuring change in social communication skills over time, including change due to interventions and therapies. The Current version asks parents to indicate the presence of a behavior within the past 3 months. The SCQ-Current has been translated in Spanish, but no psychometric or validation is available from the publisher. The SCQ-Current total score was used as a secondary child outcome measure.

#### 2.3.4 Caregiver/family profiles

##### 2.3.4.1 Parenting stress index-short form

The PSI-SF is a widely used questionnaire that taps into stressors in a parent’s life as associated with child and parent factors and parent-child interactions (56). The PSI-SF contains 36 items and yields t-scores and percentiles across three domains (Parental Distress, Parent-Child Dysfunctional Interaction, and Difficult Child) and a Total Stress score. The PSI-SF domains demonstrate good internal consistency (α = .88 -.95) and the overall Total Stress score has a strong correlation with the more comprehensive PSI-4 (*r* = .98). The Spanish version of the PSI-SF also has similar metrics across domains for internal consistency (α = .83-.90) (57, 58). The PSI-SF Total Stress score served as a primary parent outcome.

##### 2.3.4.2 Family outcomes scale-revised

In this study, we defined family empowerment as caregivers’ perceived “competence, confidence, and ability to care for their children with special needs and to achieve a satisfactory level of family adaptation” (59, 60). The FOS-R has been developed by the Early Childhood Technical Assistance Center as a family outcome measure for IDEA Part C early intervention programs in the United States. The FOS-R has been adopted by at least 20 states as of 2020-2021. The FOS-R measures family knowledge and skills across multiple areas, including Understanding child’s strengths and needs; Knowing child’s rights and advocating for child; Helping child develop and learn; Having support systems; and Accessing community. The FOS-R demonstrates good internal consistency as an overall measure (α = 0.90) and across the five subscales (α = 0.73 - 0.91). The Spanish version of the FOS-R also has similar psychometric properties, with Cronbach’s alpha ranging from 0.63-0.80 (61). The FOS-R Total score served as a primary parent outcome, with higher scores indicating greater empowerment.

##### 2.3.4.3 Peds QL family impact module

The Peds QL is a questionnaire that measures family functioning and the impact of chronic health conditions on parents and families (62). The Family Functioning section was collected and consisted of 8 questions tapping into the impact of the child’s health on daily activities (e.g., family activities taking more time and effort) and family relationships (e.g., Difficulty solving family problems together). The Peds QL is reliable and valid in capturing the quality of life among families of children with special health care needs and was available in Spanish. Family functioning was considered a secondary family outcome, with higher scores indicating better functioning.

#### 2.3.5 Intervention strategies

##### 2.3.5.1 Intervention strategy efficacy

This measure was previously developed by Magaña et al. to measure caregiver’s self-efficacy or confidence in the use of strategies covered in the Parents Taking Action program (37). For this study, additional items were added to cover additional strategies not previously covered in the Parents Taking Action program for a total of 16 items. Caregivers rated each item on a 4-point Likert scale ranging from (1) “Strongly Disagree” to (4) “Strongly Agree.” Sample items include: “*I feel confident modeling for my child what I want him/her to do*,” and “*I feel confident in using communication temptations to motivate my child to communicate*.” All items were summed, with higher scores indicating higher levels of self-efficacy in implementing the strategies. The Cronbach’s alpha was 0.68 for this scale. This measure was available in Spanish.

##### 2.3.5.2 Intervention strategy use

This measure was also previously developed by Magaña et al. to measure caregivers’ frequency in using the intervention strategies (37). Additional items were added to cover the breadth of strategies covered by the ASPEN Intervention Program, for a total of 18 items. Caregivers rated how often they implemented each strategy using a 4-point Likert scale ranging from (1) Never to (4) Always. Sample items include: “*Follow your child’s lead*” and “*Take turns with your child when you play together*.” All items were summed, with higher scores indicating greater frequency in the use of intervention strategies. The Cronbach’s alpha was 0.81 for this scale. This measure was available in Spanish.

##### 2.3.5.3 Social validity

This measure was previously adapted by Magaña et al. (63) from the Treatment Acceptability Rating Form (64) to capture the feasibility and acceptability of Parents Taking Action. The measure captures participants’ acceptability of the overall intervention and intervention strategies, and the effectiveness of the strategies for supporting the child and family (e.g., Given the learning needs and developmental delays of my child, I found an acceptable intervention strategy.). Additional items were added to this measure to capture components of the ASPEN Intervention Program that were not reflected in the versions used by Magaña et al. to evaluate Parents Taking Action (63) for a total of 18 items. Response options ranged from Strongly Disagree (1) to Strongly Agree (6). Higher scores indicated greater social validity of the ASPEN Intervention. Caregivers completed the social validity at the conclusion of the post-assessments. This measure was available in Spanish.

### 2.4 Assessment procedures

Caregivers completed the battery of assessments at baseline, after completing the intervention program, and again 3–4 months after completing the intervention program for follow-up. All assessments were completed via Zoom or over the phone with caregivers and administered by a trained research assistant. All assessments and study procedures were administered in English or Spanish based on the caregiver’s preferred language. Research assistants were blind to the family’s randomization status throughout the baseline assessment and post-intervention assessments. We implemented a communications policy across all assessments and scheduling of sessions to include 10 attempts before discontinuing a family from the study due to an inability to complete study commitments. This would include 10 attempts across phone calls, text messages, and email correspondence, with at least two attempts per week. This policy was to provide ample opportunities for families to continue their participation in the ASPEN Intervention Program. When the last attempt was reached, families were told that we would no longer be reaching out to them but that they could reach back out to the research assistant, clinician, or the study PI, if they were interested in continuing the program.

All procedures were initially planned to be implemented via in-person home visits with the child and caregivers to address challenges in low-resource households (e.g., transportation, childcare) and to support generalizability of intervention strategies in the child’s natural environment (65). However, due to the COVID-19 pandemic and the subsequent social isolation mandates worldwide starting in March 2020, in-person home visits were neither feasible nor safe for families or for our research team (29, 66). Due to these reasons, we modified our approach to complete all assessments and to provide the ASPEN Intervention Program remotely using Zoom (assessments, intervention group) or phone calls (assessments, comparison group). Caregivers who did not have a personal laptop or tablet or reliable internet were provided with a tablet and a Wi-Fi hotspot for use during their participation in the ASPEN Intervention program.

### 2.5 Intervention procedures

#### 2.5.1 Intervention group descriptions and assignments

Child-caregiver dyads were randomized into the Intervention Group or the Comparison Group upon starting the baseline assessments and stratified by the caregiver’s primary language. Both groups were designed to be completed within 12 weeks. The Intervention Group would receive 12 sessions, 1 session per week. The Comparison Group would receive 4 check-ins, 1 check-in every 3 weeks. This structure would allow similar engagement periods for families in both groups.

##### 2.5.1.1 Intervention group

Dyads who participated in the Intervention Group were assigned to a clinician and a peer leader (i.e., parent of a child with a disability). They then received 12 weekly sessions over Zoom following the structure outlined in **Table 1**. Each session was guided by a PowerPoint presentation that outlined the main points from the session, provided opportunities for discussion, and included examples of content through audio and video formats. The peer leader was present during the initial two sessions and the final two sessions to provide opportunities for the caregiver to ask questions and learn from the peer leader’s lived experience as they started and completed the program. Each session was designed to last about an hour and to be highly interactive between the parent, clinician, and peer leader (Sessions 1-2, 11-12). Psychoeducational components of the ASPEN intervention included content on stress and its impact on health and well-being, steps for relaxation and managing stress, including development and engagement with social support networks (Session 1). Additional psychoeducational components included information about child development, developmental milestones, and how to monitor for developmental delays. This was accompanied by information on how developmental delays might affect children’s daily interactions and engagement with others (e.g., communication, play, behavior). All sessions began with a brief check-in on the previous session’s home activity (e.g., practicing the strategy with the child during a daily routine, like bath time). This was followed up with an introduction to the topic, including discussions on how it relates to the child and the parent. The strategy was then discussed, including the rationale for why the strategy works to address a challenge (e.g., turn-taking). Activities were included in some sessions, where parents answered questions or filled in notes in their manuals (e.g., how their child communicates needs and wants). There were also discussion prompts about the child’s behavior, communication, or play as it relates to the topic, as well as discussions on how the strategy might be helpful for their child. Sessions 5 through 10 included individualized coaching by the clinician. The caregiver was asked to play or engage with their child in a routine or activity for at least 15 minutes (within the hour-long session) to practice the strategy that was covered in the current session. The clinician provided encouragement and feedback during or immediately after the play in the session on how to apply the strategy across daily routines. The child was only engaged during these 15-minute play segments. All sessions ended with a plan for a home activity where the parent identified a daily routine during which they would practice implementing the strategy with their child. This plan also identified a date and time for the home activity, with clinician guidance as needed. Clinicians and peer leaders were matched to child-caregiver dyads by preferred language (i.e., English, Spanish).

**Table 1:** Overview of ASPEN intervention program topics.

ASPEN intervention session topic	Intervention group	Comparison group	Adapted from:
1. Taking Care of Yourself a. ASPEN Program Introduction b. What is stress? c. How to reduce stress? d. Building a social support network	Session with Clinician and Peer Leader	Check-in with Clinician	PTA
2. Foundations of Development and Learning a. Understanding child development b. Daily routines and activities c. Setting up home for success	Session with Clinician and Peer Leader		Project ImPACT PTA
3. Foundations of Social Interactions and Play a. Stages of play development b. Following your child’s lead c. Imitating your child	Session with Clinician		Project ImPACT New content
4. Foundations of Communication Development a. What is communication? b. Communication difficulties in children with developmental delays c. Learning two or more languages d. Other communication supports	Session with Clinician	Check-in with Clinician	Project ImPACT New content
5. Creating Playful Interactions a. Playing with your child b. Animation c. Interrupting play d. Turn taking	Session with Clinician		Project ImPACT New content
6. Providing Support for Communication Development a. Model language and communication b. Expand your child’s language and communication c. Language and communication during daily routines	Session with Clinician		Project ImPACT New content
7. Creating Opportunities for Communication a. Communication temptations	Session with Clinician	Check-in with Clinician	Project ImPACT PTA
8. Introduction to Behavioral Strategies a. Overview of behavioral strategies b. Using prompts effectively c. Using reinforcement effectively d. How to use behavioral strategies	Session with Clinician		Project ImPACT PTA
9. Teaching Expressive Communication a. Teaching expressive communication b. How to prompt language skills	Session with Clinician		Project ImPACT
10. Teaching Social Imitation and Play a. Teaching social imitation b. Teaching play skills c. How to prompt play skills	Session with Clinician	Check-in with Clinician	Project ImPACT PTA
11. Addressing Challenging Behavior a. What are challenging behaviors? b. Finding the reasons for your child’s behavior c. New behaviors to reduce challenging behavior	Session with Clinician and Peer Leader		PTA New content
12. Overview of ASPEN Program a. Putting it all together b. Planning for a best possible future c. Updating goals for your child and family	Session with Clinician and Peer Leader		PTA New content

##### 2.5.1.2 Comparison group

Dyads in the Comparison Group were assigned to a clinician and received four phone-call check-ins, each about 3 weeks apart. Each check-in was designed to provide an overview of the content for three sessions (Sessions 1-3, 4-6, 7-9, 10-12), tips on how to apply the strategies in the home, and to provide the caregiver with an opportunity to ask questions. The clinician was provided a script for each check-in to ensure that they covered the main points for each session and to highlight examples to support learning and transfer of information. As part of these check-ins, clinicians also guided caregivers through the specific activities in the program manual (e.g., questions about their child’s play) and ended the session with a plan for a home activity for the caregiver to practice the strategies reviewed. Caregivers could also request additional resources at each session and check-in. Common requests included information about potty training and additional visual supports.

#### 2.5.2 Intervention materials

All families received a comprehensive manual, a resource packet that included visual supports (i.e., visual schedule, first-then board, I want … board, choice board, token economy board with tokens, and functional communication cards), Learn the Signs, Act Early materials (developmental milestones booklet, flyer for CDC Developmental Milestones mobile app, child book), and a guide on how to navigate disability services in Texas (e.g., process for requesting a school evaluation, contact information for early childhood intervention, contact information for local intellectual and developmental disability authority for their region). All materials and sessions were administered in either English or Spanish, depending on the caregiver’s preferred language. Additional details regarding the development, adaptation, and pilot trials of the ASPEN Intervention program can be found in previously published work (35, 36).

#### 2.5.3 Intervention team training and supervision

Clinicians were current advanced undergraduate and graduate students who had prior experience working with children with autism and developmental delays and their families. Disciplinary backgrounds for the clinicians included: speech-language pathology, special education, applied behavior analysis, social work, school psychology, and clinical psychology. Peer leaders were mothers of children and youth with autism or other developmental disabilities. They also had demonstrated leadership in their local communities, such as volunteering as a family advocate or participating in community advisory boards.

Clinicians and peer leaders completed over 20 hours of training on the intervention program materials and background information on autism, developmental milestones, evidence-based practices, and culturally responsive approaches in working with families from low-resource communities. The initial training sessions for clinicians and peer leaders were held in person in a group format and provided an extensive review of the intervention materials, coaching strategies, discussions on rapport building with families in low-resource communities, modeling of an intervention session, role-playing activities where clinicians practiced delivery of intervention and check-in sessions, and strategies on how to work in a team with peer leaders/clinicians. Subsequent training sessions for new clinicians and peer leaders included watching videos of the initial training content (e.g., overview of the ASPEN program and materials, developmental milestones, evidence-based practices, culturally responsive approaches). This was followed up with a virtual group session with current and new clinicians to review clinicians’ responsibilities, guidance on working with peer leaders, and dedicated time to practice and role-play the delivery of intervention sessions and check-in sessions. Feedback was provided by the PI, along with tips and strategies from current clinicians who had been working directly with families.

Clinicians met with the PI (Vanegas) weekly for group supervision and discussion of intervention implementation. This included discussions of any concerns raised by parents, questions regarding the intervention procedures, and review of intervention strategies. Clinicians also shared tips and strategies with each other on what worked and did not work for them in the sessions (e.g., examples of child activities). To monitor fidelity of implementation and family engagement, clinicians completed a fidelity checklist and parent engagement form after every session. Peer leaders met monthly with the project coordinator to discuss their participation in intervention sessions and to address any participant requests for resources.

### 2.6 Data analysis

Data were analyzed using IBM SPSS 31.0. We first examined demographic and outcome variables for missing values and group differences. No differences were observed in missingness across groups, and baseline values were not significantly associated with missingness. We then examined the data for assumptions of normality of model residuals, homogeneity of variance, and homogeneity of regression slopes. No meaningful violations of model assumptions were observed. For all analyses, we implemented a complete case analysis, using all available data elements for each analysis. This process has been deemed as efficient and robust when missing data are only present in the outcome variables (67).

To examine the effects of the ASPEN Intervention Program on the Intervention and Comparison group, we conducted an analysis of covariance (ANCOVA) controlling for baseline scores. This would allow us to measure any change across groups while accounting for baseline differences across outcomes. We applied a Bonferroni-corrected alpha of.017 (i.e.,.05/3 primary outcomes) to account for multiple comparisons. We retained *p* <.05 as the significance level for secondary outcomes as they were considered exploratory.

Due to the small sample size per group, we also conducted sensitivity analyses with paired sample t-tests to evaluate whether there were significant changes between baseline and post-intervention across all primary and secondary measures. Sensitivity analyses can provide additional tests for the robustness of findings. These analyses would allow us to examine whether there were significant changes within each group (68). This is in contrast to our primary ANCOVA analyses, which compares the degree of change across groups. We report partial η^2^ for effect sizes (small = .01, medium -.06, large = .14) and Cohen’s *d* and confidence intervals for effect sizes, with interpretation as follows: *d* ≥ 0.8 large effect, *d* = 0.5-0.79 medium effect, and *d* < 0.20–49 small effect (69).

#### 2.6.1 *Post-hoc* power analysis

We conducted a *post-hoc* power analysis using G*Power software to determine whether we were adequately powered to detect observed effects at an alpha level of.05 for all tests (70). For our main primary significant finding (intervention strategy efficacy), with a sample size of *n* = 39 for the outcome variable, and the obtained effect size of *f* = 0.39 (partial η^2^ = 0.134) with two covariates (baseline score, weeks to complete the intervention), the achieved power was 0.66. This is lower than the conventional threshold for 0.80 (69), suggesting the study was underpowered to detect significant effects. Additional *post-hoc* power analyses were also conducted with primary and secondary outcomes, showing insufficient power to detect significant effects (observed power = .05 -.22) for the remaining primary and secondary outcomes. Thus, our final sample was underpowered to detect large effects and may help inform future studies on sample size. Given that effect size estimates are independent of sample size, we highlight the effect size estimates to support the interpretation of the magnitude of effects.

### 2.7 Ethical and legal considerations

This study was registered in ClinicalTrials.gov (Identifier: NCT04505488). IRB review and approval were granted by Texas State University and the University of Texas at Austin. All participating families provided informed consent prior to their enrollment and baseline assessments.

## 3 Results

### 3.1 Analysis of sample recruitment and enrollment

**Figure 1:**
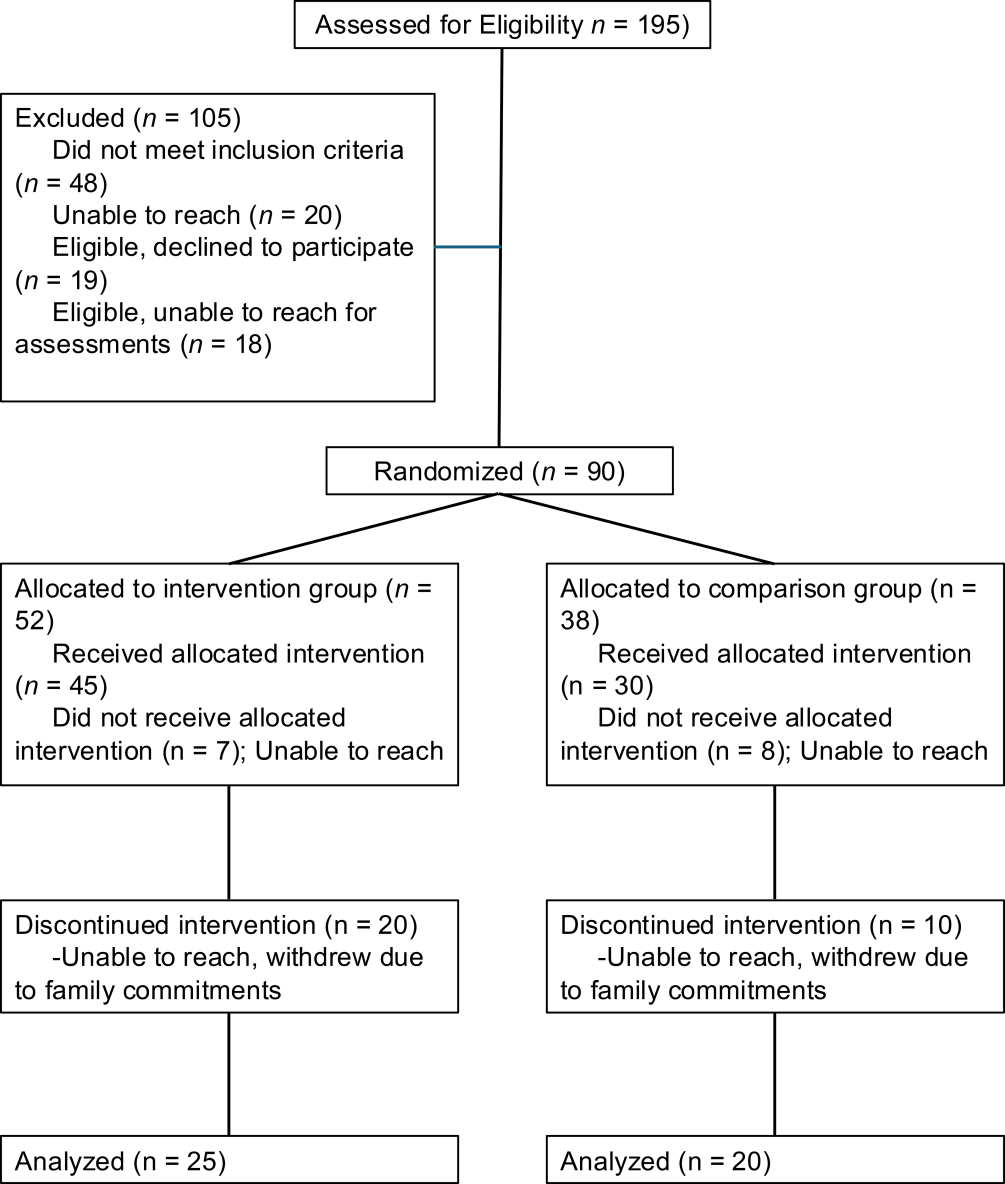
Consort diagram showing the flow of participants in the ASPEN Intervention Program.

**Figure 1** illustrates the participant flowchart for the study, adhering to the CONSORT 2025 guidelines (71). Overall, we received 195 interest forms, of which 48 were determined to be ineligible for participation and 20 were undetermined due to our inability to reach for eligibility screening (e.g., disconnected phone numbers, incomplete contact information, or no response after 10 communication attempts). We determined that 127 caregiver-child dyads were eligible to participate; however, 19 caregivers declined to participate (e.g., lack of interest, preference for child-focused intervention, time commitments, or preference for in-person program), and we were unable to reach an additional 18 to schedule the baseline assessments due to disconnected phone numbers, or no response after 10 communication attempts.

Ninety caregiver-child dyads consented and enrolled in the study and were randomized into the intervention group (*n* = 52) or the comparison group (*n* = 38). Of those who were randomized, 45 caregiver-child dyads completed at least one session from the intervention group, and 30 caregiver-child dyads completed at least one session from the comparison group. Reasons for non-completion of at least one session included being dropped from the study due to no responses to at least 10 communication attempts to schedule the session (*n* = 9) or withdrawing from the study due to family commitments (e.g., loss of job, starting a new job, limited availability; *n* = 6). Among caregiver-child dyads who completed at least one session, we had an additional 20 dyads from the intervention group and 10 dyads from the comparison group that were discontinued due to an inability to complete study commitments or withdrew from their respective assignments. No harms or unintended events were reported by any participants who enrolled in the study.

Overall, the retention rate, defined as dyads who completed the intervention after enrollment, regardless of condition, was 50%. By group, retention rates were 48% for the intervention group and 53% for the comparison group. Attrition rates were similar across both groups, χ²(1, *n* = 90) = 0.18, *p* = .831, Cramer’s *V* = .05 (small effect). No group differences emerged between dyads who completed the intervention and dyads who did not complete the intervention across child age (*p* = .892), child sex (*p* = .631), child diagnostic status (*p* = .538), caregiver age (*p* = .190), caregiver race/ethnicity (*p* = .332), education status (*p* = .389), employment status (*p* = .365), primary language (*p* = 1.00), or household income (*p* = .343).

### 3.2 Intervention fidelity

Clinicians self-reported fidelity of intervention implementation for every session. The fidelity checklist was adapted from the Project ImPACT program to support the continuity of materials. Clinicians reported on 15 behaviors (e.g., Clinician reviews progress/comments from previous session; Clinician addresses key points from the strategies/information discussed) and whether they were implemented, not implemented, or not applicable. A total fidelity score was calculated based on the total number of behaviors observed divided by the total number of behaviors possible (e.g., removing not applicable items). Average fidelity was 99.3% for the intervention group sessions and 98.5% for check-in sessions (comparison group). No significant group differences were found in fidelity of implementation across groups (*p* = .306).

### 3.3 Caregiver engagement

Clinicians rated caregiver engagement after each intervention session or check-in to measure caregiver participation with the session discussions and activities. Three questions focused on 1) active listening, 2) asking questions, and 3) active participation in discussions/activities, with ratings from 1 (not at all) to 4 (very), with higher scores indicating greater engagement. Average engagement for caregivers across their respective sessions was 3.73 (0.29) for the intervention group and 3.71 (0.45) for the comparison group. No significant group differences were found in caregiver engagement across groups (*p* = .875).

### 3.4 Demographic comparisons

Most families in the study identified as Latino, having high school or lower education, and income less than 200% of the poverty line. About 60% reported Spanish as their primary language. We compared demographic characteristics between children and caregivers in the intervention and comparison groups using independent samples t-tests and chi-square analyses. Overall, both groups were similar across most demographic and participation characteristics, except for caregivers’ time in the United States and the duration of the ASPEN Intervention Program. Although caregivers in the intervention group (*M* = 15.72 years, *SD* = 12.86 years) did not differ from caregivers in the comparison group (*M* = 22.35 years, *SD* = 14.33 years) in the number of years lived in the United States [*t*(17.73) = -2.37, *p* = .110, Cohen’s *d* = 0.49], these results indicate a small effect size. Caregivers in the intervention group also took longer to complete the ASPEN Intervention Program (*M* = 25.82 weeks, *SD* = 18.50 weeks) than caregivers in the comparison group (*M* = 15.08 weeks, *SD* = 11.92 weeks), *t*(43) = 2.25, *p* = .030, Cohen’s *d* = 0.67, indicating a medium effect size. Group means and comparisons are shown in **Table 2**.

**Table 2:** Demographic characteristics of participants who completed the ASPEN intervention program.

Participant Characteristics	Intervention group n = 25 M (SD)/% (n)	Comparison group n = 20 M (SD)/% (n)	P value	Effect size^d^
Child Characteristics				
Age in years	4.17 (1.66)	3.91 (1.38)	.581	-.17
Sex			.525	.12
Male	64.0% (16)	75.0% (15)		
Female	36.0% (9)	25.05% (5)		
Nativity (% Born in the US)	100.0% (25)	95.0% (19)	.444	.17
Race/Ethnicity^a^			1.000	.14
Latino/Hispanic	96.0% (24)	100.0% (20)		
White, Non-Latino/Hispanic	0.0% (0)	0.0% (0)		
Black/African American, Non-Latino/Hispanic	4.0% (1)	0.0% (0)		
Diagnoses (Any diagnosis)	80.0% (20)	100.0% (20)	.056	.32
Autism	68.0% (17)	90.0% (18)	.147	.26
Developmental Delay	12.0% (3)	20.0% (4)	.682	.11
Speech Delay or Language Impairment	20.0% (5)	20.0% (4)	1.000	.00
Down Syndrome	4.0% (1)	0.0% (0)	1.000	.14
ADHD	4.0% (1)	5.0% (1)	1.000	.02
Other Diagnoses^b^	12.0% (3)	25.0% (5)	.435	.17
Age when first noticed developmental differences (years)	1.28 (0.97)	1.46 (0.70)	.485	.22
Age of Diagnosis (years)	3.18 (1.33)	2.79 (1.19)	.342	-.32
Uses verbal or spoken language	76.0% (19)	55.0% (11)	.205	.22
Received early childhood intervention services	64.0% (16)	60.0% (12)	1.000	.04
Number of services currently receiving	2.48 (1.50)	2.30 (1.34)	.678	-.13
Quantity of services per week (minutes)	129.38 (144.24)	273.78 (620.38)	.343	.33
Caregiver and Family Characteristics				
Age in years	37.56 (5.44)	37.37 (7.92)	.923	-.03
Sex^c^			-–	-–
Male	0.0% (0)	0.0% (0)		
Female	100.0% (25)	100.0% (20)		
Nativity (%Born in the US)	24.0% (6)	25.0% (5)	1.000	.01
Years Lived in the US	15.72 (12.86)	22.35 (14.33)	.110	.49
Race/Ethnicity^a^			.309	.23
Latino/Hispanic	92.0% (23)	85.0% (17)		
White, Non-Latino/Hispanic	4.3% (1)	15.0% (3)		
Black/African American, Non-Latino/Hispanic	4.3% (1)	0.0% (0)		
Education Level			.760	.09
High school or lower	56.0% (14)	65.0% (13)		
Some college or greater	44.0% (11)	35.0% (7)		
Employment Status			.275	.27
Full-Time	12.0% (3)	5.0% (1)		
Part-Time	20.0% (5)	5.0% (1)		
Not Employed	68.0% (17)	90.0% (18)		
Primary Language			1.000	.04
Spanish	64.0% (16)	60.0% (12)		
English	36.0% (9)	40.0% (8)		
Household Income			.455	.17
≤200% Federal Poverty Level	100.0% (24)	95.0% (19)		
>200% Federal Poverty Level	0.0% (0)	5.0% (1)		
Intervention Characteristics				
**Number of Weeks to Complete ASPEN Intervention Program**	**25.82 (18.50)**	**15.08 (11.92)**	**.030**	**-.67**
Social Validity Score	90.15 (7.86)	93.20 (8.91)	.350	.36

^a^ Hispanic/Latino ethnicity includes multiple race designations; ^b^Other diagnoses include epilepsy, acute necrotizing encephalitis, double chromosome disorder, mood disruptive disorder, cerebral palsy, sleep disorders, dysarthria, post-traumatic stress disorder, reactive attachment disorder, microcephaly, hearing loss, cortical visual impairment, dystonia; ^c^chi-square analyses were not conducted; ^d^Effect sizes are reported as Cohen’s d for continuous variables (*d* ≥ 0.8 large effect, *d* = 0.5-0.79 medium effect, and *d* < 0.2-.49 small) or Cramer’s V for categorical variables (V > 0.50 large effect, V = 0.30-0.49 medium effect, V = 0.10-0.29 small effect) (69). Bold values indicate a significant comparison.

### 3.5 Primary analyses: child and parent outcomes

We conducted separate ANCOVAs for each primary and secondary outcome for the study, accounting for baseline values and weeks to complete the ASPEN Intervention Program as a covariate. Overall, a medium effect was observed for intervention strategy efficacy, although the ANCOVA did not reach conventional significance thresholds, *F*(1, 35) = 5.39, *p* = .026, partial η^2^ = 0.134 after applying a Bonferroni-corrected alpha of.017 due to multiple comparisons for the primary outcomes. Review of the adjusted means shows that caregivers in the intervention group reported greater efficacy in the use of intervention strategies compared to caregivers in the comparison group. Small effects were observed between groups on family empowerment [*F*(1, 36) = 0.90, *p* = .348, partial η^2^ = .024], child problem behaviors [*F*(1, 36) = 0.72, *p* = .401, partial η^2^ = .020], family functioning [*F*(1, 35) = 1.30, *p* = .264, partial η^2^ = .041], and child social communication [*F*(1, 35) = 1.01, *p* = .322, partial η^2^ = .028], although these analyses did not reach statistical significance. Adjusted means for the outcome variables are shown in **Table 3**.

**Table 3:** Analysis of covariance results examining group outcomes accounting for baseline and weeks to complete the ASPEN intervention program.

Outcome	Intervention group adjusted mean (SE)	Comparison group adjusted mean (SE)	*F*	*p*	Partial η^2^
Primary Outcome					
Child Adaptive Behaviors (VABS-3)	66.60 (2.19)	66.57 (2.48)	0.00	.993	.000
Parent Stress (PSI-SF) Total	63.63 (4.63)	62.64 (4.90)	0.02	.887	.001
Family Empowerment (FOS-R) Total	93.83 (2.53)	97.54 (2.81)	0.90	.348	.024
Child Problem Behaviors (SIB-R)	3.89 (0.42)	3.31 (0.48)	0.72	.401	.020
Family Functioning (PedsQL)	65.53 (4.10)	58.94 (3.85)	1.30	.264	.041
Child Social Communication (SCQ-C)	17.75 (1.06)	16.08 (1.15)	1.01	.322	.028
**Intervention Strategy Efficacy**	**57.30 (1.07)**	**53.54 (1.16)**	**5.39**	**.026**	**.134**
Intervention Strategy Use	54.74 (1.68)	54.69 (1.82)	0.00	.983	.000

Means are adjusted for baseline values and covariate (weeks to complete the ASPEN Intervention Program). Lower SCQ scores are demonstrate better social communication. Bold values indicate a significant comparison.

### 3.6 Sensitivity and exploratory analyses

Paired sample t-tests were conducted within each group to examine individual-level change between baseline and post-intervention outcomes.

For the Intervention Group, we found a large effect in change between baseline and post-intervention for intervention strategy efficacy (baseline *M* = 49.71, *SE* = 1.33; post *M* = 57.33, *SE* = 1.01), *t*(20) = -4.72, *p* <.001, Cohen’s *d* = -1.41. Among our child outcomes, a medium effect was observed on the VABS-3 Community subscale, indicating significant changes in community participation (baseline *M* = 33.30, *SE* = 6.24; post *M* = 37.60, *SE* = 5.26, Cohen’s *d* = -0.46). Medium effects were also observed for the family empowerment subscale Outcome 1: Understanding your child’s strengths, needs, and abilities, (baseline *M* = 15.77, *SE* = 0.65; post *M* = 17.18, *SE* = 0.49, Cohen’s *d* = -0.52), and intervention strategy use, (baseline *M* = 49.67, *SE* = 1.79; post *M* = 54.90, *SE* = 1.64, Cohen’s *d* = -0.67). Changes between baseline and post-intervention were small to negligible and did not reach conventional standards of significance for the remaining child and caregiver outcomes among the Intervention Group (see **Tables 4**, **5**).

**Table 4:** Paired sample t-tests comparing within-group change for child outcomes.

Outcome	Group	*n*	Mean change (SD)	t	*p*	Cohen’s d	Cohen’s d confidence interval
Child Adaptive Behavior							
Overall Adaptive Behavior (VABS-3 ABC)	Intervention	19	1.00 (10.72)	0.41	.689	0.08	-0.37 – 0.53
	Comparison	15	0.67 (6.96)	0.37	.716	0.04	-0.47 – 0.55
Receptive	Intervention	19	-2.21 (13.77)	-0.70	.493	-0.14	-0.59 – 0.31
	Comparison	15	-6.13 (23.27)	-1.02	.325	-0.21	-0.71 – 0.31
Expressive	Intervention	19	-1.95 (18.67)	-0.45	.655	-0.05	-0.50 – 0.40
	Comparison	15	-1.93 (11.29)	-0.66	.518	-0.05	-0.56 – 0.45
Written	Intervention	15	-4.73 (17.34)	-1.06	.308	-0.29	-0.80 – 0.24
	Comparison	10	-1.30 (6.57)	-0.63	.547	-0.07	-0.69 – 0.55
Personal	Intervention	19	-0.79 (14.07)	-0.24	.810	-0.04	-0.49 – 0.41
	Comparison	14	-1.00 (8.24)	-0.45	.657	-0.05	-0.57 – 0.48
Domestic	Intervention	15	-4.60 (11.8)	-1.51	.153	-0.29	-0.80 – 0.23
	Comparison	10	-2.20 (10.14)	-0.69	.510	-0.09	-0.71 – 0.53
Community	**Intervention**	**15**	**-6.27 (7.61)**	**-3.19**	**.007**	**-0.46**	**-0.98 – 0.08**
	Comparison	10	-4.30 (10.29)	-1.32	.219	-0.24	-0.86 – 0.4
Interpersonal	Intervention	19	0.63 (17.25)	0.16	.875	0.03	-0.42 – 0.48
	Comparison	14	-3.43 (10.8)	-1.19	.256	-0.19	-0.71 – 0.34
Play	Intervention	19	-2.63 (16.36)	-0.70	.492	-0.13	-0.58 – 0.32
	Comparison	14	-0.86 (13.16)	-0.24	.811	-0.05	-0.57 – 0.47
Coping	Intervention	19	0.68 (17.36)	0.17	.866	0.05	-0.40 – 0.50
	Comparison	13	-0.08 (7.87)	-0.04	.972	0.00	-0.55 – 0.54
Child Problem Behaviors							
Child Problem Behaviors (SIB-R) Total Count	Intervention	22	-0.50 (1.54)	-1.53	0.142	-0.21	-0.64 – 0.21
	Comparison	18	0.61 (2.17)	1.19	0.249	0.26	-0.22 – 0.72
Internalized Maladaptive Behaviors	Intervention	22	0.27 (13.38)	0.10	0.925	0.02	-0.40 – 0.44
	**Comparison**	**18**	**-12.94 (15.85)**	**-3.47**	**0.003**	**-0.99**	**-1.55 – -0.41**
Asocial Maladaptive Behaviors	Intervention	22	4.77 (15.37)	1.46	0.160	0.33	-0.11 – 0.75
	Comparison	18	0.39 (21.69)	0.08	0.940	0.03	-0.44 – 0.49
Externalized Maladaptive Behaviors	Intervention	22	2.77 (10.59)	1.23	0.233	0.17	-0.25 – 0.59
	Comparison	18	0.78 (14.17)	0.23	0.819	0.04	-0.42 – 0.51
General Maladaptive Behaviors	Intervention	22	2.73 (11.15)	1.15	0.264	0.17	-0.26 – 0.59
	Comparison	18	-3.44 (14.5)	-1.01	0.328	-0.22	-0.69 – 0.25
Child Social Communication							
Total Score (SCQ-C)	Intervention	21	-0.1 (5)	-0.09	0.931	-0.01	-0.44 – 0.41
	**Comparison**	**18**	**2.67 (4.34)**	**2.61**	**0.018**	**0.40**	**-0.09 – 0.87**

Bold values indicate a significant comparison.

**Table 5:** Paired sample t-tests comparing within-group change for caregiver outcomes.

Outcome	Group	n	Mean change (SD)	t	p	Cohen’s d	Cohen’s d confidence interval
Parenting Stress							
Total Stress (PSI-SF Percentile)	Intervention	20	6.80 (18.38)	1.65	.115	0.31	-0.14 – 0.75
	Comparison	18	10.28 (23.37)	1.87	.079	0.43	-0.06 – 0.91
Parental Distress	Intervention	20	5.50 (23.56)	1.04	.310	0.18	-0.26 – 0.62
	Comparison	18	8.50 (27.44)	1.31	.206	0.26	-0.21 – 0.73
Parent-Child Dysfunctional Interaction	Intervention	20	9.25 (23.63)	1.75	.096	0.49	0.02 – 0.95
	Comparison	18	10.28 (24.60)	1.77	.094	0.50	0.00 – 0.98
Difficult Child	Intervention	20	2.75 (19.06)	0.65	.526	0.12	-0.32 – 0.56
	Comparison	18	5.22 (19.62)	1.13	.275	0.22	-0.25 – 0.68
Family Empowerment							
Overall (FOS-R Total)	Intervention	22	-6.36 (15.60)	-1.91	.069	-0.39	-0.82 – 0.04
	**Comparison**	**18**	**-12.17 (12.59)**	**-4.10**	**<.001**	**-0.82**	**-1.35 – -0.27**
Understanding your child’s strengths, needs, & abilities	Intervention	22	-1.41 (3.47)	-1.90	.071	-0.52	-0.96 – -0.07
	**Comparison**	**18**	**-3.72 (3.44)**	**-4.59**	**<.001**	**-1.39**	**-2.04 – -0.73**
Knowing your rights and advocating for your child	Intervention	22	-2.18 (5.34)	-1.92	.069	-0.36	-0.79 – 0.08
	**Comparison**	**18**	**-2.39 (3.81)**	**-2.66**	**.016**	**-0.51**	**-1.00 – -0.01**
Helping your child to develop and learn	Intervention	22	-0.59 (2.56)	-1.08	.291	-0.21	-0.63 – 0.22
	Comparison	18	-2.61 (3.68)	-3.01	.008	-0.87	-1.40 – -0.31
Having support systems	Intervention	22	-0.77 (5.40)	-0.67	.509	-0.14	-0.56 – 0.28
	**Comparison**	**18**	**-2.78 (4.91)**	**-2.40**	**.028**	**-0.55**	**-1.04 – -0.05**
Accessing the community	Intervention	22	-1.41 (4.77)	-1.39	.180	-0.27	-0.69 – 0.16
	Comparison	18	-0.67 (3.50)	-0.81	.430	-0.17	-0.63 – 0.30
Family Functioning							
Family Functioning (PedsQL)	Intervention	16	-8.59 (22.05)	-1.56	.140	-0.40	-0.90 – 0.12
	Comparison	18	-4.51 (14.82)	-1.29	.214	-0.18	-0.64 – 0.29
Intervention Strategies							
Efficacy	**Intervention**	**21**	**-7.62 (7.39)**	**-4.72**	**<.001**	**-1.41**	**-2.01 – -0.79**
	**Comparison**	**18**	**5.11 (7.05)**	**-3.08**	**.007**	**-0.99**	**-1.54 – -0.41**
Use	Intervention	21	-5.24 (11.93)	-2.01	.058	-0.67	-1.13 – -0.19
	**Comparison**	**18**	**-4.50 (9.02)**	**-2.12**	**.049**	**-0.63**	**-1.13 – -0.12**

Bold values indicate a significant comparison.

For the Comparison Group, we found large effects in change between baseline and post-intervention for the following child and caregiver outcomes: child internalized maladaptive behaviors, (baseline *M* = -26.78, *SE* = 3.47; post *M* = -13.83, *SE* = 2.66, *t*(17) = -3.47, *p* = .003, Cohen’s *d* = -0.99; family empowerment total, (baseline *M* = 84.11, *SE* = 3.62; post *M* = 96.28, *SE* = 3.36, *t*(17) = -4.10, *p* <.001, Cohen’s *d* = -0.82; family empowerment subscale Outcome 1: Understanding your child’s strengths, needs, and abilities, (baseline *M* = 13.44, *SE* = 0.66; post *M* = 17.17, *SE* = 0.60, *t*(17) = -4.59, *p* <.001, Cohen’s *d* = -1.39.), family empowerment subscale Outcome 3: Helping your child to develop and learn (baseline *M* = 14.61, *SE* = 0.86; post *M* = 17.22, *SE* = 0.51, *t*(20) = -3.01, *p* = .008, Cohen’s *d* = -0.87); intervention strategy efficacy, (baseline *M* = 48.39, *SE* = 1.33; post *M* = 53.50, *SE* = 1.10, *t*(17) = -3.08, *p* = .007, Cohen’s *d* = -0.99).

Medium effects were observed among the following caregiver outcomes: parenting stress parent-child dysfunctional interaction subscale, (baseline *M* = 75.44, *SE* = 4.60; post *M* = 65.17, *SE* = 5.08, Cohen’s *d* = 0.50), family empower subscale Outcome 2: Knowing your rights and advocating for your child, (baseline *M* = 16.67, *SE* = 1.15; post *M* = 19.06, *SE* = 1.06, Cohen’s *d* = -0.51); family empowerment subscale Outcome 4: Having support systems, (baseline *M* = 16.06, *SE* = 1.24; post *M* = 18.83, *SE* = 1.14, Cohen’s *d* = -0.55); and intervention strategy use (baseline *M* = 50.00, *SE* = 1.69; post *M* = 54.50, *SE* = 1.67, Cohen’s *d* = -0.63). Small to negligible effects were evident for the remaining child and caregiver outcomes among the Comparison Group.

Overall, we conducted 93 statistical tests. We completed comparisons across 27 demographic variables to describe and characterize our sample. We ran 8 statistical tests across primary and secondary outcomes, with Bonferroni correction applied to the 3 primary outcomes. An additional 16 tests were part of our sensitivity analyses to examine the robustness of the findings. Finally, 42 tests were exploratory analyses of subtests/subscales within the primary and secondary outcomes. There is the possibility that some of the significant findings reflect chance findings due to multiple comparisons and should be interpreted with caution.

## 4 Discussion

In this study, we tested the efficacy of the ASPEN Intervention Program, a culturally adapted, parent-mediated intervention designed for racially/ethnically diverse families in low-resource households. Of those who completed the study, we found meaningful differences in intervention strategy efficacy (confidence) for caregivers in the Intervention Group, even after accounting for baseline levels of efficacy and the length of time to complete the intervention program. Caregivers in the intervention group received a comprehensive manual and 12 weekly remote sessions with the student clinicians that included psychoeducation and coaching with their child. Intervention group caregivers improved their efficacy (or confidence) in using intervention strategies and their reported use of those strategies. Other studies of PMIs have also found significant effects on caregiver efficacy (72, 73). It may be that the additional coaching that caregivers in the intervention group received provided that additional support and confidence in implementing evidence-based strategies at home with their child. Additional analyses found medium effects in changes between baseline and post-intervention on family empowerment (understanding your child’s strengths, needs, and abilities), intervention strategy use, and child adaptive behavior in community (children’s daily functioning in settings outside of their home, including understanding safety, concepts of money, and travel). This suggests that our current study may be underpowered.

Caregivers in the comparison group received all the materials from the intervention and monthly check-in calls with clinicians. Sensitivity analyses examining within group change found several large effects for the comparison group. Specifically, caregivers reported a large decrease in internalized maladaptive behavior in their children, as well as large increases in overall family empowerment and within family empowerment domains (understanding your child’s strengths, needs, and abilities; helping your child develop and learn). Large effects were also observed on intervention strategy efficacy. Other RCT studies of PMIs have also reported significant gains across both intervention and control groups, suggesting that for some families, receiving any intervention may improve outcomes (74, 75). When we tested for group differences, we found that efficacy in using strategies was stronger for the intervention group caregivers at post-intervention than it was for the comparison group.

These findings may reflect the challenges for families with low resources to engage in more frequent regular intervention (76). It took caregivers in the intervention group on average 25 weeks to complete the 12-week intervention, which is more than twice the time intended. In contrast, caregivers in the comparison group were able to complete the tri-weekly check-ins in 15 weeks, on average. For these caregivers, it may be that having the materials and monthly meetings may go a long way to addressing some of their training needs given other demands on their time. These findings are similar to those found in other studies of families with limited resources (41, 65, 77). When implementing a parent education program to low-income Black families, Dababnah and colleagues found that only 3 out of 7 mothers completed all 12 weeks of the program, and there were many cancellations and missed appointments (41). Those who did not complete the program reported benefiting from the sessions they did attend. A study by Carr and Lord with families in low-resource households also reported a high attrition rate of 62% among families who enrolled in their parent-mediated intervention project (65). For this latter study, participation in the six-month intervention took almost nine months for some families due to frequent cancellations.

Our recruitment flow chart also demonstrates challenges families with low resources may have in engaging in a weekly intervention. Out of 127 parents who expressed interest and were eligible to participate, only 90 enrolled in the study. Furthermore, only half of those who enrolled in the study completed their participation. This is similar to a second study by Dababnah of low-income Black parents (46), in which a little more than half completed the study. Two studies, one of Navajo mothers of children with autism reported, and the other of low-income Latino mothers reported better rates of retention, but were still relatively low at about 70% (77, 78). Furthermore, several studies of parenting interventions, some of which were implemented in low-resource communities, have also reported similar challenges with participant engagement and retention (32, 33, 79–81).

### 4.1 Limitations

As previously described (36), the ASPEN Intervention Program was launched in the initial months of the COVID-19 pandemic. The unprecedented difficulties that communities faced, especially low-resource communities, created significant challenges for the engagement, recruitment, and retention of families. Despite the contextual difficulties, additional limitations may have affected the outcomes of this study. The ASPEN Intervention Program was initially developed for in-person home sessions as past research shows that this approach is most effective for families in low-resource households (82). However, given the circumstances of COVID-19, in-person home visits were not available. Relatedly, for some of the early enrolled families, our assessments may have inadvertently captured heightened stress due to the related COVID-19 challenges (e.g., lockdown, remote school, loss of services) (83, 84). This may have limited the potential effects of the ASPEN Intervention Program on reducing parenting stress, a primary caregiver outcome.

Another limitation of this study was the lack of a true control group. The substantial disparities and inequities that families of young children with autism and developmental disabilities face in accessing services prompted the need for a comparison group that could still receive some form of support. Including a no-intervention control group could be considered as unethical due to the level of need that families in low-resource households already face, especially considering the evidence base of PMIs in supporting families (85). The lack of a true control group could have masked the effects of receiving the ASPEN Intervention Program.

Finally, attrition in both groups poses a limitation for this study as the results reflect outcomes of caregivers who were available and able to complete the intervention. Although reasons for withdrawal or discontinuation from the respective groups were mostly tied to external factors (e.g., loss of jobs), there is a possibility that some families who withdrew or discontinued from the program did so in response to the acceptability or feasibility of the ASPEN Intervention Program. With a differential attrition rate of 4.5%, this study is considered to have a tolerable threat of bias, but only under optimistic assumptions (86). Thus, the results presented here should be interpreted with caution as they may be subject to attrition bias. Additional research is needed to further evaluate participant engagement and the acceptability and feasibility of the intervention program with larger samples.

### 4.2 Future directions

This study compared two delivery models that can be further tested in real-world settings. Future research should further explore the active ingredients within community settings to examine whether varying levels of the ASPEN Intervention can result in changes at the child-, caregiver-, and family-level. Larger samples across broader geographical areas can enable future studies to examine subgroups (e.g., child age groups, home language environment) and potential differential effects of the ASPEN intervention program. For example, although we included a broad age range of children, we did not have enough children across groups (e.g., 18–35 months, 36–83 months) to examine whether the ASPEN intervention program had a differential impact based on the child’s age. Furthermore, although most of the children included in the current study had been identified as having autism, future research can evaluate whether the ASPEN intervention program may also be beneficial for caregivers of children with other developmental disorders (e.g., developmental delays, Down Syndrome). Additional research is also needed to identify child, caregiver, and family characteristics that would be most responsive to parent-mediated intervention programs.

### 4.3 Conclusions

A primary finding from this study is that those in the intervention group reported greater levels of self-efficacy or confidence in using evidenced based (EB) strategies with their children than the comparison group, and both groups reported increases in using EB strategies. Theoretically, parents’ confidence and use of strategies is the first step towards positive child outcomes. Further follow-up may show the impact parents improved behaviors may have on child outcomes, however more time is likely needed to demonstrate this impact.

## Data Availability

The raw data supporting the conclusions of this article will be made available by the authors, without undue reservation.
